# Safety and effectiveness of switch to bictegravir/emtricitabine/tenofovir alafenamide following dual regimen therapy in people with HIV: Insights from the Icona cohort

**DOI:** 10.1111/hiv.70037

**Published:** 2025-04-28

**Authors:** Andrea De Vito, Alessandro Tavelli, Alessandro Cozzi‐Lepri, Andrea Giacomelli, Roberto Rossotti, Giacomo Ponta, Nicoletta Bobbio, Alice Ianniello, Antonella Cingolani, Giordano Madeddu, Andrea Antinori, Antonella d'Arminio Monforte, ICONA Foundation Study Group, A. d'Arminio Monforte, A. Antinori, S. Antinori, A. Castagna, R. Cauda, G. Di Perri, E. Girardi, R. Iardino, A. Lazzarin, G. C. Marchetti, C. Mussini, E. Quiros‐Roldan, L. Sarmati, B. Suligoi, F. Von Schloesser, P. Viale, A. d'Arminio Monforte, A. Antinori, A. Castagna, F. Ceccherini‐Silberstein, A. Cingolani, A. Cozzi‐Lepri, A. Di Biagio, E. Girardi, A. Gori, S. Lo Caputo, G. Marchetti, F. Maggiolo, C. Mussini, M. Puoti, C. F. Perno, C. Torti, A. Antinori, A. Bandera, S. Bonora, A. Calcagno, D. Canetti, A. Castagna, F. Ceccherini‐Silberstein, A. Cervo, A. Cingolani, P. Cinque, A. Cozzi‐Lepri, A. d'Arminio Monforte, A. Di Biagio, R. Gagliardini, A. Giacomelli, E. Girardi, N. Gianotti, A. Gori, G. Guaraldi, S. Lanini, G. Lapadula, M. Lichtner, A. Lai, S. Lo Caputo, G. Madeddu, F. Maggiolo, V. Malagnino, G. Marchetti, A. Mondi, V. Mazzotta, C. Mussini, S. Nozza, C. F. Perno, S. Piconi, C. Pinnetti, M. Puoti, E. Quiros Roldan, R. Rossotti, S. Rusconi, M. M. Santoro, A. Saracino, L. Sarmati, V. Spagnuolo, N. Squillace, V. Svicher, L. Taramasso, C. Torti, A. Vergori, A. Cozzi‐Lepri, S. De Benedittis, I. Fanti, M. Giotta, C. Marelli, A. Rodano, A. Tavelli, M. Cernuschi, L. Cosmaro, A. Perziano, V. Calvino, D. Russo, M. Farinella, N. Policek, V. L. Del Negro, M. Augello, S. Carrara, S. Graziano, G. Prota, S. Truffa, D. Vincenti, R. Rovito, M. Sgarlata, I. A Giacometti, A. Costantini, V. Barocci, A. Saracino, C. Santoro, E. Milano, L. Comi, C. Suardi, P. Viale, L. Badia, S. Cretella, E. M. Erne, A. Pieri, E. Quiros Roldan, E. Focà, B. Menzaghi, C. Abeli, L. Chessa, F. Pes, P. Maggi, L. Alessio, G. Nunnari, B. M. Celesia, J. Vecchiet, K. Falasca, A. Pan, S. Dal Zoppo, D. Segala, F. Bartalesi, C. Costa, S. Lo Caputo, S. Ferrara, M. Bassetti, E. Pontali, S. Blanchi, N. Bobbio, C. Del Borgo, R. Marocco, G. Mancarella, S. Piconi, C. Molteni, S. Rusconi, G. Canavesi, G. Pellicanò, G. Marchetti, S. Antinori, G. Rizzardini, M. Puoti, A. Castagna, A. Bandera, V. Bono, M. V. Cossu, A. Giacomelli, R. Lolatto, M. C. Moioli, L. Pezzati, S. Diotallevi, C. Tincati, C. Mussini, M. Menozzi, P. Bonfanti, G. Lapadula, V. Sangiovanni, I. Gentile, V. Esposito, N. Coppola, F. M. Fusco, G. Di Filippo, V. Rizzo, N. Sangiovanni, S. Martini, A. M. Cattelan, D. Leoni, A. Cascio, M. Trizzino, D. Francisci, E. Schiaroli, G. Parruti, F. Sozio, D. Messeri, S. I. Bonelli, C. Lazzaretti, R. Corsini, A. Antinori, R. Cauda, C. Mastroianni, L. Sarmati, A. Latini, A. Cingolani, I. Mastrorosa, S. Lamonica, M. Capozzi, M. Camici, I. Mezzaroma, M. Rivano Capparuccia, G. Iaiani, C. Stingone, L. Gianserra, J. Paulicelli, M. M. Plazzi, G. d'Ettore, M. Fusto, M. Lichtner, I. Coledan, G. Madeddu, A. De Vito, M. Fabbiani, F. Montagnani, A. Franco, R. Fontana Del Vecchio, D. Francisci, C. Di Giuli, G. C. Orofino, G. Calleri, G. Di Perri, S. Bonora, G. Accardo, C. Tascini, A. Londero, G. Battagin, S. Nicolè, G. Starnini, S. Dell'Isola

**Affiliations:** ^1^ Unit of Infectious Disease, Department of Medicine, Surgery and Pharmacy University of Sassari Sassari Italy; ^2^ PhD School in Biomedical Science, Biomedical Science Department University of Sassari Sassari Italy; ^3^ ICONA Foundation Milan Italy; ^4^ National PhD Programme in One Health Approaches to Infectious Diseases and Life Science Research, Department of Public Health, Experimental and Forensic Medicine University of Pavia Pavia Italy; ^5^ Centre for Clinical Research, Epidemiology, Modelling and Evaluation (CREME), Institute for Global Health University College London London UK; ^6^ III Infectious Diseases Unit ASST FBF‐Sacco Milan Italy; ^7^ Department of Infectious Diseases ASST Grande Ospedale Metropolitano Niguarda Milan Italy; ^8^ Infectious Diseases Unit, IRCCS San Raffaele Scientific Institute Vita Salute San Raffaele University Milan Italy; ^9^ Department of Infectious Diseases Galliera Hospital Genoa Italy; ^10^ Division I of Infectious and Tropical Diseases ASL Città di Torino Turin Italy; ^11^ Clinic of Infectious Diseases, Department of Safety and Bioethics Università Cattolica del Sacro Cuore Rome Italy; ^12^ National Institute for Infectious Diseases, Lazzaro Spallanzani IRCCS National Institute for Infectious Diseases, Lazzaro Spallanzani IRCCS Rome Italy

**Keywords:** ART switch, bictegravir, dual therapy, viral load

## Abstract

**Objectives:**

Most treatment switches are for simplification from three‐drug (3DR) to dual regimens (2DR). However, a proportion of people with HIV may switch back to 3DR, like bictegravir/emtricitabine/tenofovir alafenamide (B/F/TAF) after 2DR.

**Methods:**

We included people with HIV enroled in the Icona cohort who switched to B/F/TAF after 2DR INSTI‐based (3TC/DTG, RPV/DTG, RPV/CAB, or DOR + DTG). Virological rebound (VR), virological suppression (VS), and treatment discontinuation (TD) due to toxicity or failure were evaluated using Kaplan–Meier curves. Random intercept and slopes before and after the switch were used to evaluate the trajectories of triglycerides, cholesterol, CD4, and CD4/CD8. Viro‐immunological analyses were stratified according to HIV‐RNA at switch.

**Results:**

Among the 3662 people with HIV who started a 2DR INSTI‐based regimen, 71 (1.9%) switched to B/F/TAF; 60 had been followed up after the switch, for a median of 10.9 months (interquartile range: 3.6–24.7). Forty people with HIV switched with HIV‐RNA <50 copies/mL (uVL), 20 with HIV‐RNA ≥50 copies/mL (dVL). Among the uVL group, one participant experienced VR (HIV‐RNA: 99, 71 followed by 29 copies/mL). Among the dVL group, the 1‐year cumulative probability of undetectable VL was 75% (95% confidence interval [CI] 57.6–95.1). Fourteen people with HIV interrupted B/F/TAF for simplification (50.0%), toxicity (28.6%), VR (14.2%), and patient's choice (7.1); the 1‐year cumulative probability of TD for toxicity/failure was 10.7% (95% CI 14.5–24.5). We observed an increase in the CD4/CD8 ratio (+0.02 CD4/CD8/month, *p* = 0.026) only in the dVL group.

**Conclusions:**

Switching from 2DR‐INSTI to B/F/TAF is infrequent; this switch results in a low rate of toxicity and failure, along with a favourable immunovirological and lipid profile. CD4/CD8 gain is observed in those switching with detectable HIV‐RNA.

## BACKGROUND

The prevention or management of comorbidities or pharmacologic interactions, the need to reduce the number of pills and/or daily doses, and the onset or prevention of toxicities are some of the reasons that may lead to the optimization (i.e., improvement of overall treatment outcomes through adjustments in regimen composition) of antiretroviral therapy (ART) in people with HIV [1, 2]. However, maintaining a stable virologic suppression is a prerequisite for treatment optimization. In the current HIV treatment landscape, a two‐drug regimen (2DR) based on the combination of dolutegravir represents a relevant option in clinical practice [1, 2, 3]. Specifically, the 2DRs available on the market are lamivudine (3TC)/DTG and rilpivirine (RPV)/DTG [4, 5, 6].

Currently, these two options represent a key strategy for simplification according to EACS and NIH international guidelines [1, 2]. In addition, in the last years that it became available, the long‐acting intramuscular injection (LAI) combination of cabotegravir (CAB) plus RPV became available every 4–8 weeks [7, 8]. Finally, another possible combination reported in many studies is the combination of doravirine (DOR) and DTG [9]. The real‐life data showed that the percentage of treatment discontinuation of 2DR is probably higher than that shown in clinical trials [10, 11]. RCTs of bictegravir co‐formulated with emtricitabine and tenofovir alafenamide (B/F/TAF) were shown to be non‐inferior to DTG‐containing 3DR in virologically suppressed adults [12] and B/F/TAF, as recommended by international guidelines, is widely used in real life as a switched regimen among individuals with undetectable viral load, with good effectiveness also in key groups like elderly people with HIV [13]. Therefore, transitioning to a 3DR, such as the single‐tablet regimen of B/F/TAF, may be warranted to ensure sustained virologic suppression and effectively manage other clinical issues.

However, while many trials and real‐life studies have evaluated the switch from triple to dual therapy, focusing on the efficacy, safety, and durability of these 2DRs, there are only anecdotal data in the literature regarding the outcomes of people with HIV who switch from a 2DR to a single‐tablet 3DR regimen.

The aim of this retrospective analysis is to characterize individuals who were switched from 2DR DTG‐based or CAB/RPV back to a 3DR with B/F/TAF in clinical practice in Italy in order to identify the drivers of switch, to evaluate the virologic and immunologic response as well as the lipid changes following the switch to B/F/TAF.

## METHODS

### Study design

We conducted a retrospective analysis of prospectively collected cohort data, including individuals enrolled in the ICONA Foundation cohort. The ICONA study is a prospective observational study, established in 1997, that enrols people with HIV who are naïve to ART at inclusion. It includes reasons for treatment discontinuation, which are systematically collected and updated biannually during routine follow‐up visits. Currently, the ICONA database includes more than 21 500 people with HIV enrolled in 62 centres across Italy, and participants are considered representative of the average people with HIV receiving care in Italy. Further details of the ICONA Study are reported elsewhere [14].

### Study population

In this study, we included all ICONA people with HIV enrolled from 2019 to 2024 who were switched back to a 3DR regimen with B/F/TAF after a period of treatment with oral or injectable 2DR‐based integrase inhibitors (INSTI): 3TC/DTG, RPV/DTG, long‐acting RPV/CAB, or DOR + DTG, regardless of HIV‐RNA at the switch to B/F/TAF. We included only patients that switched to 2DR while virologically suppressed. The time of switch to B/F/TAF was the time zero for all prospective analyses (baseline).

### Objectives

The *primary objectives* were to estimate the cumulative probability of (i) treatment discontinuation (TD) of B/F/TAF for toxicity or failure, (ii) virological rebound (VR) among those undetectable at baseline, and (iii) virological suppression among those viremic at baseline.

The *secondary objectives* were to describe the reasons for switching from INSTI‐based 2DR to B/F/TAF and to evaluate the changes in CD4, CD4/CD8, and lipid profile (triglycerides, total cholesterol, low‐density lipoprotein [LDL], and high‐density lipoprotein [HDL]) after baseline.

### Statistical analyses

Demographic and clinical characteristics of the people with HIV included in the study were described by means of absolute and relative frequency for categorical data and median with interquartile range (IQR) for quantitative variables. Reasons for switching from 2DR to B/F/TAF, and the reasons for interruption of B/F/TAF were also reported as absolute numbers and relative frequencies.

Kaplan–Meier curves were used to investigate the cumulative 1‐year probability of: (a) time to treatment discontinuation for toxicity or failure of B/F/TAF, (b) time to VR on treatment defined as two consecutive HIV‐RNA ≥50 copies/mL while on B/F/TAF or a single HIV‐RNA ≥1000 copies/mL followed by a change of ART, among those with undetectable viral load (HIV‐RNA <50 copies/mL, uVL) at baseline and (III) time to virological suppression (VS) defined as reaching HIV‐RNA <50 copies/mL among those with detectable viral load (HIV‐RNA ≥50 copies, dVL) at baseline. Median survival with a 95% confidence interval (CI) was also reported for the VS endpoint.

Linear mixed models for small samples (Kenward and Roger method), with random intercepts and slopes per patient with a change in slope before and after baseline, were used to evaluate the trajectories of triglycerides, cholesterol, CD4, and CD4/CD8 ratio over the 2 years before and after baseline. In addition to the change in slope, we also tested, independently, the pre‐ and post‐baseline trajectories, whether they were different from zero. We hypothesized at the outset that the slope of immunological parameters might differ according to HIV‐RNA strata at baseline (dVL and uVL). We performed a formal interaction test and showed the results of the linear mixed model after stratifying by level of baseline HIV‐RNA.

### Ethics

The Icona Foundation study received approval from the institutional review boards of all participating centres. All patients provided written consent to participate in the study and for the processing of personal data, adhering to the ethical standards set by the committee on human experimentation and the Helsinki Declaration (last amended in October 2013). All the data are recorded and stored in a pseudo‐anonymized database to maintain confidentiality.

## RESULTS

Among the 3932 people with HIV who started a 2DR INSTI‐based regimen while virologically suppressed, 71 (1.9%) switched to B/F/TAF after a median exposure to 2DR regimens of 0.7 years (IQR: 0.4–2.1); of these, 60 (84.5%) had follow‐up data available after the switch to B/F/TAF, for a median time of 10.9 months (IQR: 3.6–24.7).

The majority of participants were cisgender males (*n* = 45, 75%) with a median age of 49 (IQR 38–57) years. At the time of switch, 40 individuals (66.7%) had undetectable HIV‐RNA (uVL, <50 copies/mL), while 20 (33.3%) had detectable HIV‐RNA (dVL, ≥50 copies/mL), including 13 with low‐level viremia (50–200 copies/mL). Among the 20 people with HIV with detectable HIV‐RNA at switch, a genotypic resistance test (GRT) was performed for 11. No patients developed resistance mutations to INSTI after the failure (Table S1).

Most people with HIV who initiated B/F/TAF (*n* = 42, 75%) were previously treated with either 3TC/DTG or 3TC plus DTG, followed by those switching from long‐acting CAB + RPV (*n* = 13, 21%). Baseline characteristics of the 60 people with HIV included in the analysis are summarized in Table 1.

**TABLE 1:** Characteristics of 60 people with HIV treated with 2DR INSTI‐based therapy who were switched to a 3DR treatment with B/F/TAF.

	B/F/TAF switch from 2DR‐INSTI (*N* = 60)
Cisgender female, *n* (%)	15 (25.0)
Age (years), median [IQR]	49 [38, 57]
Duration of previous ART (years), median [IQR]	6.8 [3.3, 10.5]
Born in Italy, *n* (%)	54 (90.0)
Modality of HIV acquisition, *n* (%)	
Heterosexual	25 (41.7)
IDU	4 (6.7)
MSM	31 (51.7)
History of AIDS‐defining pathology, *n* (%)	10 (16.7)
Zenith HIV‐RNA (log copies/mL), median [IQR]	4.8 [4.2, 5.4]
Nadir CD4 (cells/mL), median [IQR]	294 [132, 407]
Nadir CD4 (cells/mL), *n* (%)	
<200	18 (30.0)
<350	39 (65.0)
Year of B/F/TAF start, median [IQR]	2022 [2021, 2023]
Number of previous regimens, median [IQR]	4 [3, 6]
Dual regimens before switch, *n* (%)	
3TC/DTG or 3TC + DTG	42 (70.0)
RPV + CAB	13 (21.7)
DOR + DTG	1 (1.7)
RPV/DTG or RPV + DTG	4 (6.7)
Duration of 2DR (years), median [IQR]	0.7 [0.4, 2.1]
CD4 (cells/mL) at baseline, median [IQR]	612 [465, 861]
CD8 (cells/mL) at baseline, median [IQR]	774 [578, 1096]
CD4/CD8 at baseline, median [IQR]	0.70 [0.58, 1.12]
HIV‐RNA (copies/mL) at baseline, *n* (%)	
<50	40 (66.7)
50–200	13 (21.7)
>200	6 (11.7)
Total cholesterol (mg/dL) at baseline, median [IQR]	183 [154, 216]
LDL (mg/dL) at baseline, median [IQR]	115 [92, 147]
HDL (mg/dL) at baseline, median [IQR]	46 [39, 60]
Triglycerides (mg/dL) at baseline, median [IQR]	115 [74, 153]

Abbreviations: 2DR, two‐drug regimen; 3TC, lamivudine; ART, Antiretroviral therapy; B/F/TAF, bictegravir/emtricitabine/tenofovir alafenamide; baseline, B/F/TAF start; CAB, cabotegravir; DOR, doravirina; DTG, dolutegravir; HDL, high‐density lipoproteins; IDU, intravenous drug users; IQR, inter quartile range; LDL, low‐density lipoproteins; MSM, men who have sex with men; RPV, rilpivirine.

The majority of participants were cisgender males (*n* = 45, 75%), with a median age of 49 (IQR, 38–57) years. Forty (66.7%) people were switched with undetectable HIV‐RNA (uVL, <50 copies/mL), and 20 (33.3%) with detectable HIV‐RNA (dVL, ≥50 copies/mL), and (13/20 with HIV‐RNA of 50–200 copies/mL). The majority of people with HIV (*n* = 42, 75%) who started B/F/TAF were previously treated with 3TC/DTG or 3TC + DTG, followed by the long‐acting CAB + RPV (*n* = 13, 21%). The baseline characteristics of the 60 people with HIV included in the analyses are summarized in Table 1.

### Reasons for switching

The main reasons for switching to B/F/TAF were: 28.3% (*n* = 17) failure of the 2DR regimen, 18.3% (*n* = 11) pill reduction, 16.3% (*n* = 10) patients' choice, 21.7% (*n* = 13) toxicities, and 15% (*n* = 9) other/unknown reasons. Toxicities included neuropsychiatric symptoms (*n* = 5), reactions at injection sites for CAB/RPV LAI formulation, allergic reactions (*n* = 2 each), arthromyalgia, gastrointestinal intolerance, renal toxicity, and clinical contraindications (*n* = 1 each).

### Virological response

**FIGURE 1:**
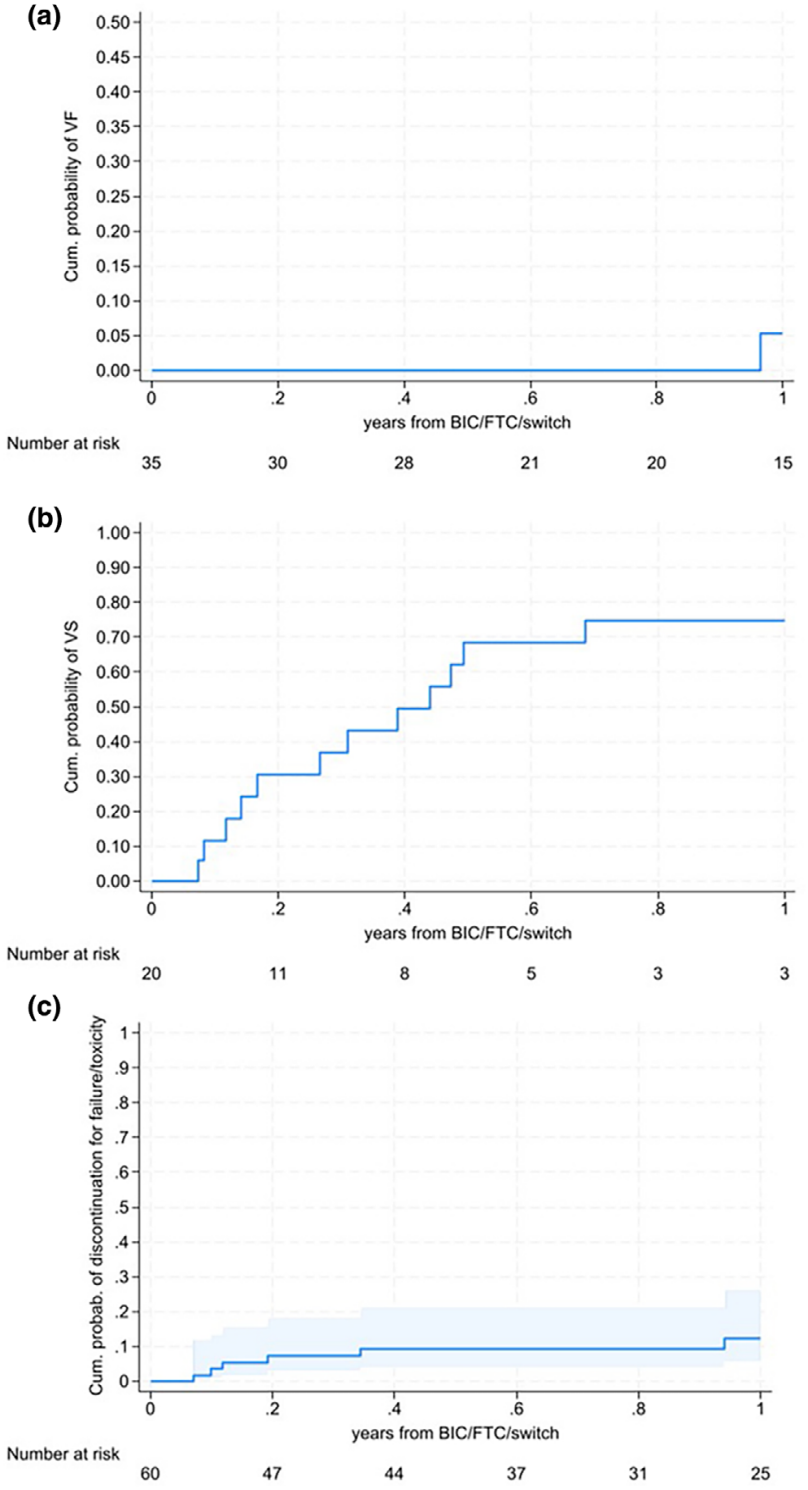
(a) Kaplan–Meier curves of the cumulative probability of virological rebound (VR) after bictegravir/emtricitabine/tenofovir alafenamide (B/F/TAF) switch in virologically suppressed people with HIV at baseline; (b) Kaplan–Meier curves of the cumulative probability of virological suppression (VS) after B/F/TAF switch in people with HIV with detectable HIV‐RNA at baseline; (c) Kaplan–Meier curves of the cumulative probability of TD of B/F/TAF for toxicity or failure.

Among those who were switched to B/F/TAF with uVL (<50 copies/mL), with at least one virological follow‐up available on B/F/TAF (*n* = 35), only one virological failure was reported 11.6 months after the B/F/TAF switch. It was a low‐level viremia rebound with HIV‐RNA 99 and 71 copies/mL in two consecutive values. The patient subsequently reached stable VS without the need to switch away from B/F/TAF. The cumulative probability of VR at 12 months in the whole sample of people with HIV with baseline uVL was, therefore, 5.2% (95% CI 0.8–31.9) (Figure 1).

Among those who were switched to B/F/TAF with a dVL and with available virological follow‐up (*n* = 20), the 1‐year cumulative probability of achieving VS was 74.8% (95% CI 52.6–92.2), while the median survival time to reaching VS was 0.44 years (95% CI 0.14–0.68) (Figure 1b).

### B/F/TAF discontinuation

Overall, 14 people with HIV (23.3%) interrupted B/F/TAF, half of them (*n* = 7) returned to 2DR for simplification (5 on 3TC/DTG and 2 on LAI CAB/RPV); one discontinued B/F/TAF for participant's choice (7.1%) and switched to FTC/TAF/RPV; two for failure (14.2%—1 switched to the multi tablet regimen FTC/TAF + DTG and one to a complex regimen of 3TC + DTG + DOR); and finally four (28.6%) for toxicity (1 for weight gain, 1 for neuropsychiatric adverse event, 1 for tachycardia, and 1 for unspecified toxicity). The regimens started after B/F/TAF interruption for toxicity were 2 FTC/TAF/RPV, 1 FTC/TAF + DOR, and 1 FTC/TAF + DRV/c.

The 1‐year cumulative probability of TD of B/F/TAF for toxicity or failure was 10.7% (95% CI 0.5–24.5) (Figure 1c).

### CD4 and CD4/CD8 ratio changes

We had evidence for an interaction between period pre/post baseline and HIV‐RNA at baseline only for CD4/CD8 ratio (*p* = 0.035) and not for CD4 cell count (*p* = 0.740); therefore, we reported the CD4/CD8 ratio trajectories stratified for baseline HIV‐RNA (dVL vs. uVL), and the CD4 cell count slope overall without stratification. We observed an increase in the CD4/CD8 (+0.02 CD4/CD8 per month, 95% CI 0.003–0.044, *p* = 0.024) during B/F/TAF only in people with HIV who switched with a detectable viral load. However, there was no evidence for a change in the slope of CD4/CD8 after the switch to B/F/TAF compared with pre‐baseline (slope difference post‐ vs. pre‐baseline = +0.02 CD4/CD8/month; 95% CI −0.006 to 0.045; *p* = 0.126). There is also no evidence for a difference in slope after the switch compared with the pre‐baseline in CD4/CD8 ratio and in CD4 cell count for people with HIV with uVL (Table S2).

### Lipid profile

Regarding the lipid profile, we observed no evidence for a change in the slope of total cholesterol, triglycerides, and LDL after baseline compared with pre‐baseline and a slight increase of HDL (−0.410 mg/dL/month, 95% CI 0.023–0.7986, *p* = 0.038) (Table S3). None of the patients initiated a lipid‐lowering drug after the switch.

## DISCUSSION

Previous studies showed that the switch from 2DR to 3DR is infrequent and mainly due to failure of the 2DR regimen [15, 16, 17]. To our knowledge, ours is the first analysis focusing on people with HIV who were switched from dual INSTI‐containing to B/F/TAF in a real‐world setting. In our cohort, including more than 3500 people with HIV on dual INSTI‐containing regimens, only 71 (1.9%) switched back to B/F/TAF.

Our analysis confirms that the majority (33%) were switched because of virological failure and another 20% because of toxicity. Interestingly, 18% switched because of pill reduction. DTG/3TC has been available as one pill in Italy only since the end of 2019, and this may explain the request for pill reduction as a reason to switch to a single‐tablet triple regimen.

Among participants who were virologically suppressed at the time of switching to B/F/TAF, we observed only one case of HIV‐RNA transitory low‐level rebound over a 1‐year follow‐up; viral load returned to an undetectable level without changing therapy.

Notably, among those with detectable viral loads at the time of the switch, 74.8% achieved virologic suppression within 1 year, highlighting B/F/TAF's role in re‐establishing control over viral replication in this group of people with HIV failing ART. Overall, a 1‐year viral control <90% can be considered suboptimal, but without a proper comparison, it is indeed difficult to draw conclusions, thus considering this selected population that failed dual therapy DTG based.

Our results are similar to those reported by Chang et al., who evaluated people who were switched to B/F/TAF from integrase inhibitors, protease inhibitors, or non‐nucleoside reverse transcriptase inhibitors 3DR because of virological failure [15]. They reported a 92% virological suppression rate after switching to B/F/TAF, with a median time to suppression of 12 weeks. Among those with detectable viral load prior to starting BIC/FTC/TAF (*n* = 27, 15.4%), 81.5% achieved and maintained virological suppression during follow‐up, a higher proportion than that observed in our study (74.8%). It is important to note, however, that in the study by Chang et al., only seven participants were receiving INSTI‐based regimens at the time of virological failure, which may account for the higher suppression rates reported in their cohort. These findings underscore B/F/TAF's versatility and effectiveness in diverse clinical scenarios. Additionally, in people who started B/F/TAF with a detectable viral load, we observed an increase in CD4/CD8 ratio, even if there was no evidence for a difference in slope compared with pre‐baseline and clinical impact remains to be established.

Regarding the metabolic profile, we observed a slight increase in HDL levels, with a mean change of 0.41 mg/dL/month (95% CI 0.023–0.796; *p* = 0.038). No evidence of a change in slope was noted for total cholesterol or low‐density lipoprotein (LDL) levels and triglycerides. These findings align with existing literature on the metabolic effects of B/F/TAF [15, 17].

Our study has several limitations that should be considered when interpreting the findings. Firstly, the retrospective nature may introduce biases related to data collection and accuracy, as it relies on existing records that were not initially intended for this research purpose. Secondly, with only 60 participants, the study's statistical power is limited, and the wide CIs observed in some outcomes reflect this constraint. This small sample size is, however, partly inevitable as switches from dual INSTI‐based regimens to B/F/TAF in real life are infrequent. Third, rates of VS and VR of B/F/TAF could only be compared with those previously reported in the literature as we did not include a control group of people with HIV of Icona who were switched to B/F/TAF without previously experiencing a period of treatment with 2DR. Finally, we don't have data for all people with HIV about blood pressure and weight to assess.

## CONCLUSIONS

The intensification of dual INSTI‐containing regimens to B/F/TAF was infrequent in our cohort and primarily (45% cases) occurred in individuals experiencing virological failure on dual therapy, which is itself a rare event. Nevertheless, the switch to B/F/TAF resulted in a successful outcome both in terms of viro‐immunological and clinical findings. B/F/TAF's efficacy and tolerability make it a compelling option for treatment optimization, even in individuals experiencing challenges with 2DRs.

## AUTHOR CONTRIBUTIONS

*Conception*: Andrea De Vito, Antonella d'Arminio Monforte, Alessandro Tavelli, Andrea Antinori and Giordano Madeddu. *Study design*: Andrea De Vito, Antonella d'Arminio Monforte, Alessandro Tavelli and Alessandro Cozzi‐Lepri. *Accessing and verifying data*: Alessandro Tavelli and Andrea De Vito. *Statistical analysis*: Alessandro Tavelli. *Acquisition of data*: Andrea Giacomelli, Roberto Rossotti, Giacomo Ponta, Nicoletta Bobbio and Alice Ianniello. *Patients' enrolment*: Andrea De Vito, Andrea Giacomelli, Roberto Rossotti, Giacomo Ponta, Nicoletta Bobbio, Antonella Cingolani and Giordano Madeddu. *Draft of the manuscript*: Andrea De Vito, Antonella d'Arminio Monforte and Alessandro Tavelli. *Review of the article and critical revision for important intellectual content*: All authors. *Reading and final approval of the submitted version*: All authors.

## FUNDING INFORMATION

This project was realized with the support of Gilead Sciences, who provided an unrestricted grant. The funder had no role in the conception, data collection, analysis, and interpretation of results. The Icona Foundation is supported by unrestricted grants from Gilead Sciences, ViiV Healthcare, Merck Sharp & Dohme, and Janssen‐Cilag.

## CONFLICT OF INTEREST STATEMENT

Alessandro Tavelli, Alessandro Cozzi‐Lepri, Alice Ianniello, Antonella d'Arminio Monforte, Nicoletta Bobbio, Giacomo Ponta declare no conflicts of interest. Andrea De Vito received fees for attending advisory boards from ViiV. Andrea Giacomelli received fees for attending advisory boards from ViiV. Speakers' honoraria from Gilead, Janssen, MSD, and ViiV. Roberto Rossotti received grants, fees for speaker's bureau, and advisory board activities from ViiV Healthcare, MSD, Gilead Sciences, Johnson & Johnson, and SD Biosensor. Antonella Cingolani received funding for scientific advisory boards, travel, or speaker honoraria from Gilead Sciences, ViiV Healthcare, Janssen‐Cilag, and MSD. Giordano Madeddu received speaker's honoraria and fees for attending advisory boards from Viiv, Gilead, MSD, Janssen, and Tera Technologies. Andrea Antinori served as a paid consultant to Astra Zeneca, Bavarian Nordic, Gilead Sciences, GSK, Janssen‐Cilag, MSD, Moderna, Pfizer, and ViiV Healthcare and received institutional research grants from Astra Zeneca, Gilead Sciences, and ViiV Healthcare.

## Supporting information


**Data S1.** Supporting information.

## Data Availability

The datasets generated during the current study are not publicly available because they contain sensitive data to be treated under data protection laws and regulations. Appropriate agreement of data sharing can be arranged after a reasonable request to the corresponding author.
