# Supplementary material for: Safety and effectiveness of switch to bictegravir/emtricitabine/tenofovir alafenamide following dual regimen therapy in people with HIV: Insights from the Icona cohort
Source: HIV Med. 2025 Apr 28;26(6):970–7. doi: 10.1111/hiv.70037 (PMC12127237; doi:10.1111/hiv.70037)
Supplement: Supplementary file 1 — Data S1. Supporting information. [file HIV-26-970-s001.docx]

**Supplementary Material**

**Table 1-S**. Genotype Resistance Test performed after virological failure to two drugs regimen.

| **Number** | **HIV-RNA** | **note** | **data** | **Subtype** | **NRTI mutations** | **NNRTI mutations** | **PI mutations** | **INSTI mutations** | **BIC** | **FTC** | **TAF** |
| --- | --- | --- | --- | --- | --- | --- | --- | --- | --- | --- | --- |
| 1 | 178 | Performed | 13/09/2023 | B | M184V | none | none | none | S | HR | S |
| 2 | 78 | Not Performed | . |  |  |  |  |  |  |  |  |
| 3 | 73 | Not Performed | . |  |  |  |  |  |  |  |  |
| 4 | 88 | Not Performed | . |  |  |  |  |  |  |  |  |
| 5 | 60 | Performed but not amplifiable RT and PI | 30/07/2021 | . | not amplifiable | not amplifiable | not amplifiable | none | S | . | . |
| 6 | 66500 | Performed | 16/09/2023 | B | none | K101KE,E138EA | none | E157Q | S | S | S |
| 7 | 144 | Performed | 27/04/2022 | A | none | none | none | none | S | S | S |
| 8 | 144 | Performed | 11/02/2022 | A1 | none | none | none | none | S | S | S |
| 9 | 66 | Not Performed | . |  |  |  |  |  |  |  |  |
| 10 | 143 | Performed but not amplifiable RT, PI and INSTI. | 13/09/2021 | . | not amplifiable | not amplifiable | not amplifiable | not amplifiable | . | . | . |
| 11 | 58 | Not Performed | . |  |  |  |  |  |  |  |  |
| 12 | 178000 | Performed | 27/02/2023 | B | none | none | none | none | S | S | S |
| 13 | 304 | Performed but not amplifiable RT and PI.* | 08/11/2022 | CRF02_AG | not amplifiable | not amplifiable | none | not amplifiable | . | . | . |
| 14 | 32305 | Performed | 18/03/2021 | B | none | none | none | none | S | S | S |
| 15 | 110 | Not Performed | . |  |  |  |  |  |  |  |  |
| 16 | 90 | Not Performed | . |  |  |  |  |  |  |  |  |
| 17 | 35020 | Performed | 28/10/2023 | B | M184I | none | L10V, L63A (other) | none | S | HR | S |
| 18 | 172 | Performed but not amplifiable RT, PI and INSTI. | 30/07/2020 | . | not amplifiable | not amplifiable | not amplifiable | not amplifiable | . | . | . |
| 19 | 159000 | Not Performed | . |  |  |  |  |  |  |  |  |
| 20 | 209 | Not Performed | . |  |  |  |  |  |  |  |  |
| * Another test was performed during a blip on TAF/FTC/BIC, and no mutation was detected on RT and INSTI. | | | | | | | | | | | |

**Table 2S.** *Mean changes/month of CD4 and CD4/CD8 from fitting linear mixed models with change at B/F/TAF switch (baseline)*

|  |  | **2-years pre-baseline** | | **2-years post-baseline** | | | **Contrast slopes pre/post baseline** | |
| --- | --- | --- | --- | --- | --- | --- | --- | --- |
|  | **n** | **slope, change/month [95%CI]** | **p** | **slope, change/month [95%CI]** | **p** | **slope difference, change/month [95%CI]** | | **p** |
| **CD4 count, cells/mm3** | 57 | -0.565  [ -4.39, 3.26] | 0.772 | -1.963  [ -5.684, 1.759] | 0.301 | -1.398  [ -6.366, 3.571] | | 0.581 |
| **CD4/CD8 ratio (uVL)** | 38 | 0.000  [ -0.006, 0.005] | 0.872 | 0.003  [ -0.003, 0.008] | 0.369 | 0.003  [ -0.005, 0.011] | | 0.443 |
| **CD4/CD8 ratio (dVL)** | 19 | 0.004  [ -0.015, 0.023] | 0.685 | 0.024  [ 0.003, 0.044] | 0.024 | 0.02  [ -0.006, 0.045] | | 0.126 |

**Table 3-S.** *Mean changes of total cholesterol, HDL, LDL and triglycerides from fitting linear mixed models with change at B/F/TAF switch*

|  |  | **2-years pre-baseline** | | **2-years post-baseline** | | **Contrast slopes pre/post baseline** | |
| --- | --- | --- | --- | --- | --- | --- | --- |
|  | n | **slope, change/month [95%CI]** | **p** | **slope, change/month [95%CI]** | **p** | **slope difference, change/month [95%CI]** | **p** |
| **Cholesterol, mg/dL** | 55 | 0.349  [ -0.442, 1.141] | 0.387 | -0.269  [-1.033, 0.495] | 0.490 | -0.618  [-1.705, 0.468] | 0.265 |
| **HDL, mg/dL** | 53 | -0.212  [-0.522, 0.099] | 0.182 | 0.198  [-0.083, 0.479] | 0.167 | 0.41  [0.023, 0.796] | 0.038 |
| **LDL, mg/dL** | 50 | 0.099  [-0.692, 0.891] | 0.806 | -0.587  [-1.315, 0.141] | 0.114 | -0.686  [-1.759, 0.387] | 0.210 |
| **Triglycerides, mg/dL** | 55 | 0.106  [-1.089, 1.301] | 0.862 | -1.177  [-2.389, 0.035] | 0.057 | -1.283  [-2.95, 0.385] | 0.132 |
